# Pharmaceutical Nanoparticles Formation and Their Physico-Chemical and Biomedical Properties

**DOI:** 10.3390/ph17050587

**Published:** 2024-05-05

**Authors:** Tatyana I. Shabatina, Yana A. Gromova, Olga I. Vernaya, Andrei V. Soloviev, Andrei V. Shabatin, Yurii N. Morosov, Irina V. Astashova, Michail Y. Melnikov

**Affiliations:** 1 Department of Chemistry, M.V. Lomonosov Moscow State University, Moscow 119991, Russia; chemyaninka@mail.ru (Y.A.G.); olga_vernaya@mail.ru (O.I.V.); fa.andrey@mail.ru (A.V.S.); yunmor@mail.ru (Y.N.M.); melnikov46@mail.ru (M.Y.M.); 2 Faculty of Fundamental Sciences, N.E. Bauman Moscow Technical State University, Moscow 105005, Russia; 3 Frumkin Institute of Physical Chemistry and Electrochemistry RAN, Moscow 119071, Russia; 5dr.on5@mail.ru; 4 Department of Mechanic and Mathematics, Lomonosov Moscow State University, Moscow 119991, Russia; ast.diffiety@gmail.com

**Keywords:** drug nanoforms, electrospraying, spray drying, emulsion solvent evaporation, supercritical fluidic technology, cryochemical micronization, cryogenic freeze-drying, vacuum vapor deposition

## Abstract

The use of medicinal substances in nanosized forms (nanoforms, nanoparticles) allows the therapeutic effectiveness of pharmaceutical preparations to be increased due to several factors: (1) the high specific surface area of nanomaterials, and (2) the high concentration of surface-active centers interacting with biological objects. In the case of drug nanoforms, even low concentrations of a bioactive substance can have a significant therapeutic effect on living organisms. These effects allow pharmacists to use lower doses of active components, consequently lowering the toxic side effects of pharmaceutical nanoform preparations. It is known that many drug substances that are currently in development are poorly soluble in water, so they have insufficient bioavailability. Converting them into nanoforms will increase their rate of dissolution, and the increased saturation solubility of drug nanocrystals also makes a significant contribution to their high therapeutic efficiency. Some physical and chemical methods can contribute to the formation of both pure drug nanoparticles and their ligand or of polymer-covered nanoforms, which are characterized by higher stability. This review describes the most commonly used methods for the preparation of nanoforms (nanoparticles) of different medicinal substances, paying close attention to modern supercritical and cryogenic technologies and the advantages and disadvantages of the described methods and techniques; moreover, the improvements in the physico-chemical and biomedical properties of the obtained medicinal nanoforms are also discussed.

## 1. Introduction

The effectiveness of a drug substance is determined by its composition and its ability to dissolve and interact with a biological target. The low bioavailability of poorly soluble medicinal molecules in water is determined not only by the slow rate of penetration, but also by the kinetics of solid-phase dissolution. The rate of dissolution is directly proportional to the specific surface area of the solid drug dosage form particles. Additionally, reducing the size to the nanoscale increases the solubility of the drug in saturation [1,2]. Theoretically, this effect can be predicted by the Ostwald–Freundlich equation:

(1) $$\;ln\frac{C_{s,r}}{C_{s,\infty }}=\frac{2\gamma M}{\rho rRT}\;$$

where *C_s,r_* and *C_s,∞_* are the solubilities of a particle of radius *r* and of infinite size, *γ* is the interfacial tension between the drug particles and the solubilization medium, *M* and *ρ* are the drug particles’ molecular weight and density, *R* is the gas constant, and *T* is the absolute temperature. 

Reducing the medicinal substances particle size of from several microns to tens of nanometers results in a significant increase in their specific surface area by two orders of magnitude. This enhanced specific surface area has a profound effect on improving the bioavailability of the pharmaceutical substance nanoforms. Size is a key factor, as smaller drug particles, measuring less than 100–200 nm in size, can easily bypass the protective barriers of human and animal bodies, more effectively accumulate in tissues, and even can penetrate inside the cells of various organisms with greater efficiency [3,4,5,6,7,8,9].

Oral administration is the most common treatment approach for a wide range of diseases due to its safety, patient convenience, and cost-effectiveness. However, some drugs are ineffective when administered orally due to poor adsorption and permeability through the mucus and epithelium of the gastrointestinal tract. Structural and dimensional modification in drug nanoparticles can significantly impact their interaction with biological pathways and alter oral drug bioavailability. By adjusting the size and shape of particles, it is possible to enhance their absorption rate on the mucous membrane of the digestive system as well as their penetration into the intercellular space [4,10,11,12,13,14,15,16,17,18,19]. 

Thus, pharmaceutical nanoparticulate systems have significant potential in addressing issues such as low bioavailability and suboptimal therapeutic effects associated with delivering poorly water-soluble drugs. The submicron scale of these systems enhances the therapeutic and pharmacokinetic properties of medications, allowing a reduction in the administered dosage and a decrease in the toxicity effects. Various types of solid-drug micronization methods are employed to produce drug nanoparticles, categorized as “top-down” or “bottom-up” approaches, depending on the initial material [20,21]. In the case of “top-down” methods (such as wet milling and high-pressure homogenization), particle size reduction occurs, starting with larger solid particles that transform into nanomaterial particles. Conversely, in “bottom-up” methods (including spray drying, electrospraying, cryogenic freeze-drying, cryogenic vapor condensation, solvent evaporation, liquid anti-solvent, and supercritical antisolvent techniques), pharmaceutical particles are created through atomic and molecular level association or aggregation processes. 

## 2. Milling

Milling is a mechanical technique commonly employed in various industries for reducing particle size, frequently applied in industry. It is often used in pharmaceutical formulation. Its benefits include environmental friendliness and cost-effectiveness. During the grinding process, mechanical energy is transferred to the particles, causing them to experience stress and deformation. This stress leads to the formation and propagation of cracks, ultimately resulting in bulk material destruction. In crystalline materials, fracturing predominantly occurs along the cleavage planes of the crystals. The dispersion of drug substances is promoted by an increased concentration of crystal lattice defects, particularly dislocations. 

The process of grinding a solid substance suspended in a liquid is referred to as “wet grinding”. This method is a widely utilized technique in the contemporary pharmaceutical industry to achieve submicron drug crystals, thereby enhancing dissolution rates and oral absorption. Numerous pharmaceutical nanodrugs are produced using this technique, including Avinza^®^ (Morphine sulfate), Azopt^®^ (Brinzolamide), Emend^®^ (Aprepitant), Focalin XR^®^ (Dexmethylphenidate HCl), Herbesser^®^ (Diltiazem), Invega^®^ Sustenna^®^ (Paliperidone palmitate), Megace ES^®^ (Megestrol acetate), Naprelan^®^ (Naproxen sodium), Rapamune^®^ (Sirolimus (rapamycin)), Ritalin LA^®^ (Methylphenidate HCl), Theodur^®^ (Theophylline), Tricor^®^ (Fenofibrate), Verelan^®^ PM (Verapamil HCl), and Zanaflex^®^ (Tizanidine HCl) [22]. 

Apigenin nanoparticles were obtained in [23] using the planetary ball milling technique. Without a stabilizer, grinding in the mill led to the formation of particles of approximately 2 microns in size. After 120 min, 37% of the resulting nanosized powder was dissolved, compared to only 14% for the original pharmacopoeias form. The addition of stabilizers enabled the reduction in particle size and enhancement of dissolution rate. Captisol facilitated the generation of 1760 nm particles with 46% dissolution within 120 min. With Pluronic F127, the characteristics were 317 nm and 46% dissolution, while polyvinyl pyrrolidone (PVP) yielded 278 nm particles and 52% dissolution, respectively.

A Cilnidipine nanosuspension with an average particle size of 312 nm was developed [24] using wet milling. The suspension contained PVP as the steric stabilizer and sodium lauryl sulfate as the electrostatic stabilizer. The pharmacokinetics of the resulting nanoform were assessed in vivo using Sprague Dawley rats. The maximum drug concentration of the nanosuspension in blood plasma was approximately three times higher than for the initial bulk Cilnidipine.

An Albendazole nanosuspension obtained through wet planetary bead milling [25] with particle size of about 180 nm showed exhibited a 13.50-fold increase in the average dissolution rate compared to an unground dispersion in a medium at pH = 1.2. Furthermore, the maximum average solubility value was also enhanced to 1.45.

The nanoformulation of the poorly water-soluble drug Ketoprofen was developed using mannitol medium and a Hoover automatic miller [26]. Nanoparticles of the oral antidiabetic agent Pioglitazone were produced [27] through the wet milling technique in a planetary ball mill, resulting in particle sizes ranging from 600 to 2000 nm depending on the experimental conditions. The dissolution rate of the nanoformulation increased by 1.5 to 10 times. The impact of fabrication conditions and parameters on the particle size of Efavirenz nanosuspension is discussed in publication [28]. Additionally, milling techniques were utilized to enhance the effectiveness of the poorly soluble drug Midazolam [29].

## 3. High-Pressure Homogenization

**Figure 1:**
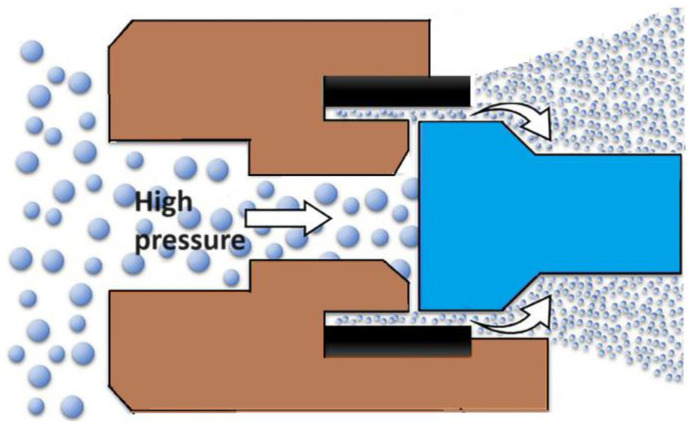
The general scheme of the drug nanoparticles production process using the high-pressure homogenization technique.

High-pressure homogenization is a technique used in the pharmaceutical industry to mix, grind, and stabilize dispersions by passing a liquid through a narrow gap under high pressure (50 to 500 MPa) (see Figure 1) [30]. One of commonly used variants of this method is a two-stage technology that combines precipitation with high-pressure homogenization. This method has been successfully used to form nanocrystals of drugs such as Albendazole [31], Carbamazepine [32], and Amphotherin B [33]. Nanosuspensions of Aprepitant and Ibuprofen have also been produced [34] using a combination of pretreatment methods, including precipitation and ball milling followed by high-pressure homogenization, resulting in nanoparticles of about 100 nm in size. The optimized nanoformulations of Ibuprofen and Aprepitant demonstrated improved solubility and dissolution profiles higher than pure drugs. Currently, this method is employed in the pharmaceutical industry to obtain nanoforms of Spironolactone, Cyclosporine, and Fenofibrate [35].

## 4. Electro Sprayed Technique 

**Figure 2:**
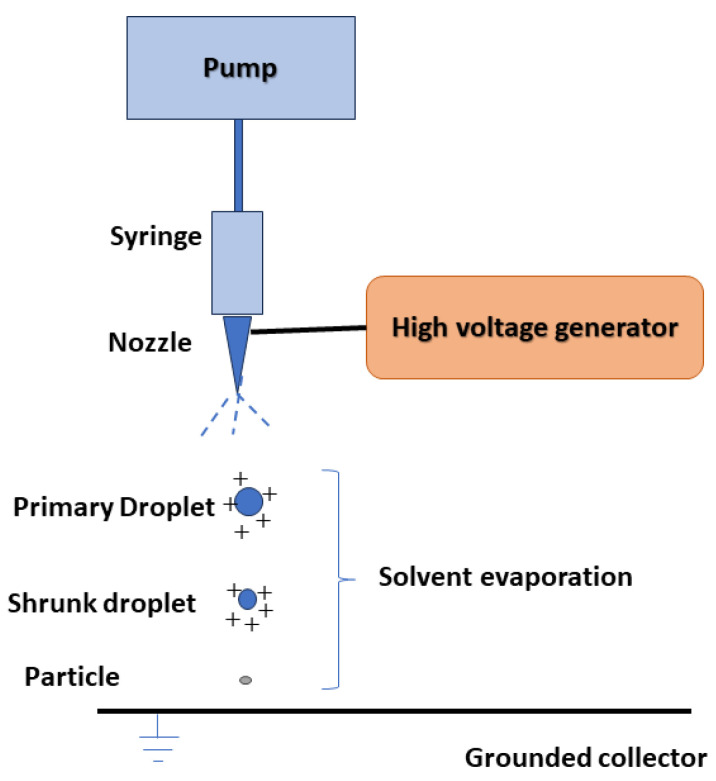
The general scheme of the drug nanoparticles production process using the hydrodynamic electrospray technique.

Drug nanoparticles can be obtained using the electro hydrodynamic atomization technique, also known as the electrospray method, which relies on electrostatic force. In this process, a precursor solution is introduced through an electrically charged nozzle opening into the collector (Figure 2). As the solution travels towards the collector, the solvent evaporates, leading to the formation of nanoparticles. By adjusting parameters such as the type and concentration of the dissolved substance, the presence of modifiers and stabilizers, voltage, flow rate, and the distance between the nozzle and the collector, particles of different sizes and morphologies can be produced [35,36]. The electrospray technique offers several advantages over other methods, including a narrow size distribution of particles, single-step operation at ambient temperature and pressure, and the absence of surfactants or stabilizers in the sprayed solution. The resulting products are dry powders with minimal residual solvent concentrations [35,36]. 

Niflumic acid nanoparticles were produced [37] using the electrospray crystallization method, with particle size and shape influenced by preparation parameters. The average crystal size increased from 300 to 3000 nm as the concentration of the precursor solutions rose from 10 to 30 mg/mL. The shape of the crystals varied based on the original drug solution concentrations, with higher concentrations yielding crystals which were more needle-shaped and lower concentrations producing crystals close to spherical-like shapes. Particle size was also affected by the potential difference and nozzle size, with smaller potential differences and larger nozzle diameters leading to crystal agglomeration. The optimal synthesis conditions identified by the authors included a solution concentration of 20 mg/mL, a potential difference of +4.7 kV, a nozzle diameter of 0.33 mm, a working distance 17 mm, and a flow rate of 1.8 mL/h, which resulted in prismatic-shape Niflumic acid crystals with an average size of approximately 500 nm.

In the case of Carbamazepine produced via the electrospray process, an increase in particle size from submicron to 6 microns was observed as the drug concentration in the original solution increased from 4 to 12 mg/mL [38]. Higher concentrations led to the formation of needle-like and tear-shaped particles, with an optimal concentration of 6 mg/mL for obtaining micron-sized needles. Further increases in concentration resulted in the formation of larger tear-shaped particles.

The drug Simvastatin, known for its extremely low solubility and low oxidative stability, was formulated as hybrid particles with polyvinyl pyrrolidone to enhance solubility and maintain chemical stability [39]. For the electrospray process, several solutions containing 4–20% (*w*/*v*) PVP and 7.5% (*w*/*v*) isopropanol were prepared. A nozzle with a diameter of 600 µm was utilized, and a positive high voltage (8.8–13.4 kV) was applied to the nozzle, while the collector was negatively charged at −3 kV. The resulting particles exhibited predominantly spherical shapes and sizes ranging from 500 to 1300 nm. However, these particles were not stable, with only 6% of the drug remaining after 30 days. The addition of butylated hydroxy anisole to the particles increased the stable drug proportion to 96%. Nonetheless, the enhancement in chemical stability led to a reduction in drug’s solubility in aqueous buffer.

Stable nanocrystal formation of Docetaxel was achieved [40] to improve its solubility. Following electrospraying, the average particle size was 458 ± 57 nm. In vivo studies further validated the enhanced therapeutic potential of electrosprayed nanocrystals, demonstrating a reduction in tumor burden compared to the drug solution.

## 5. Supercritical Technologies

**Figure 3:**
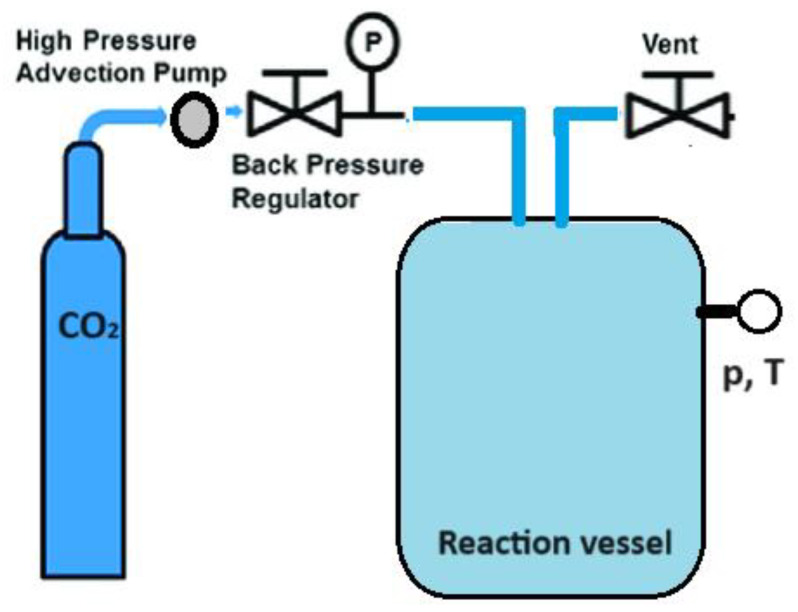
The general scheme of drug nanoparticles production process using a supercritical CO_2_-assisted technique.

Another approach to enhancing the therapeutic effectiveness of poorly soluble drugs by reducing their particle size involves the supercritical CO_2_-assisted technique (Figure 3) [41,42,43,44,45,46]. A substance reaches a supercritical fluid state when its temperature and pressure surpass the critical point. Supercritical CO_2_ with a moderate critical temperature of 31.1 °C and pressure of 7.3 MPa can be used to obtain nanomaterials with an amorphous or crystalline structure by adjusting experimental conditions such as temperature and pressure. This technique leverages the solubility properties of supercritical CO_2_, acting as a solvent for soluble solutes, and as an antisolvent for insoluble solutes. It enables the production of drug nanoforms without stabilizers or other impurities.

**Figure 4:**
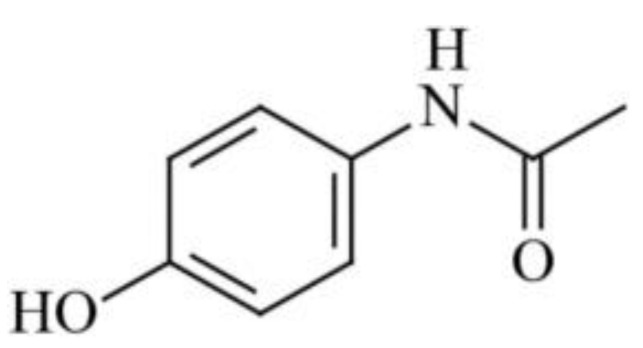
The structural formula of Acetaminophenol.

Nanoparticles of the antitumor drug Capecitabine were produced using CO_2_ as a gas antisolvent (with Dimethyl sulfoxide as the solvent) at 308 K and 160 bar [47]. This method significantly reduced the average particle size of the drug from 65 microns to 250 nm. Sertraline hydrochloride nanoparticles were synthesized [47] using Gas Anti-Solvent (115 nm) and Rapid Expansion of Supercritical Solution with Solid Cosolvent (185 nm) processes, resulting in dissolution rates that were 50 and 18 times higher than those of the original bulk preparation. Modification conditions such as extraction pressure, temperature, pre-expansion temperature, and spraying distance influenced the particle size of Acetaminophen (Figure 4), ranging from 30 microns to 12 nm [48], whereas the original pharmacopeial drug had a particle size of 85 microns.

**Figure 5:**
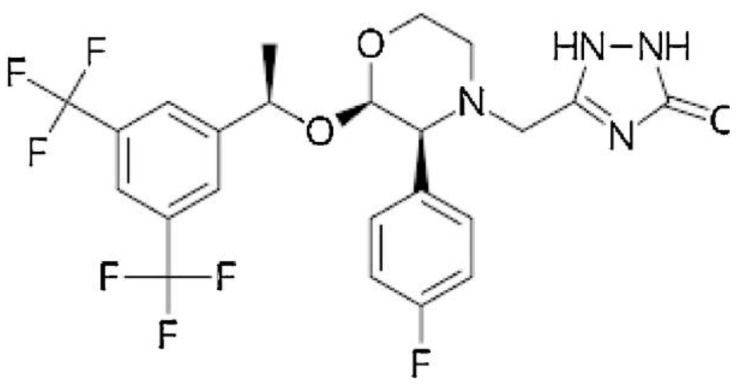
The structural formula of Aprepitant.

**Figure 6:**
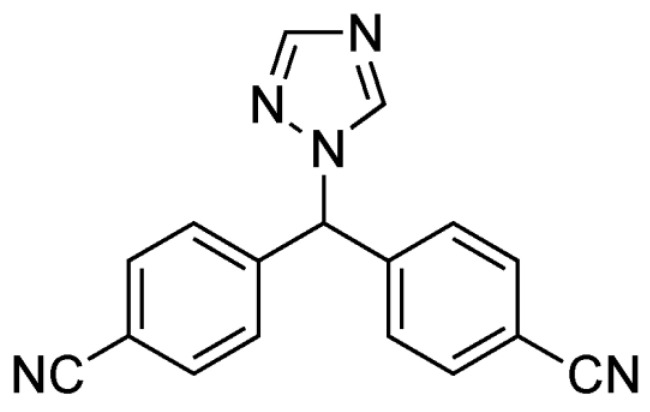
The structural formula of Letrozole.

Rapid expansion of a supercritical solution using a solid co-solvent reduced the particle size of Aprepitant (Figure 5) from 23 microns to 25 nm [49], leading to an 8.2-fold increase in dissolution rate after the supercritical process. Nanosized Letrozole (Figure 6), an anticancer drug, was produced through rapid expansion of supercritical solutions with a solid co-solvent [50], reducing the average particle size from thirty microns to tens and hundreds of nanometers. Depending on synthesis conditions, this size could range from 19 to 500 nm, with the solubility of the maximally reduced substance increasing by 7.1 times compared to the original formulation.

Supercritical technologies have been shown to reduce the particle size of Fenofibrate down to 1.8–8.33 µm compared to untreated Fenofibrate (24.2 ± 0.8 µm) [51]. Supercritical anti-solvent technology was utilized to reduce the particle size of Betamethasone [52], Myricetin [53], and Luteolin [54]. Additionally, rapid expansion of drug supercritical solution was employed to achieve 300 nm particles of Haloperidol [55].

The relatively low operating temperatures (25–40 °C) for processing pharmaceutical components and the absence of residual solvent in the final product make the supercritical approach an attractive option for the pharmaceutical industry. 

## 6. Cryogenic Technologies (Cryogenic Freeze Drying of Water Solutions and Low Temperature Vapor Condensation in Vacuum)

**Figure 7:**
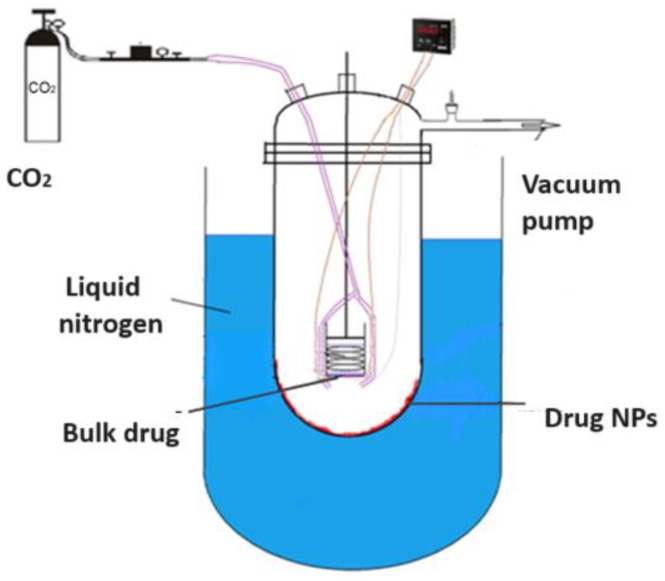
The general scheme of drug nanoparticles production process using the low temperature drug vapor condensation from gas phase.

**Figure 8:**
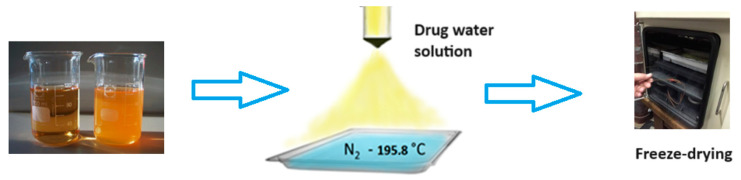
The general scheme of drug nanoparticles production process using the freeze-drying technique.

Cryochemical methods of synthesis and modification of drug nanoparticles are based on the combination of the processes of evaporation or sublimation of a drug substance by heating under vacuum conditions, followed by condensation of the drug molecular vapor at support surfaces cooled to cryogenic temperatures (77–100 K) [56,57] (Figure 7). Another powerful cryogenic technology is the spray freeze-drying technique of frozen water or mixed water–organic solutions of drug substances under vacuum conditions [56,58,59,60,61,62,63,64,65] (Figure 8). The use of low temperatures enables the reduction in drug substance particles down to the nanoscale and stabilization of metastable crystal structures, allowing the creation of new polymorphic crystal modifications of known drug substances. Low temperatures are also necessary to prevent uncontrolled transformations of intermediate and target products and to control the properties of the resulting materials. Polymorphic modification is the most important technological reserve for increasing the efficacy of drug substances [66,67]. Usually, polymorphism of a drug substance is understood as its ability to exist in two or more crystalline structures (crystal modifications), which have different structures. These structures differ in thermodynamic, mechanical, and spectral characteristics. Often these characteristics lead to significant changes in the biopharmaceutical parameters of dissolution and absorption in vitro and in vivo, stability, and properties of dosage forms. Important parameters that affect the crystallization process of polymorphic modifications of drug substances are temperature, pressure, degree of supersaturation, and nature of the solvent. Moreover, temperature and pressure play a decisive role in the formation of certain polymorphic modifications, since they determine the conditions of their stability and solubility. Let us consider specific examples of the cryochemical synthesis of nanosized drug powders [56,57,68,69,70,71,72,73,74,75,76,77,78].

### 6.1. 5-Androstenediol-3β,17β

**Figure 9:**
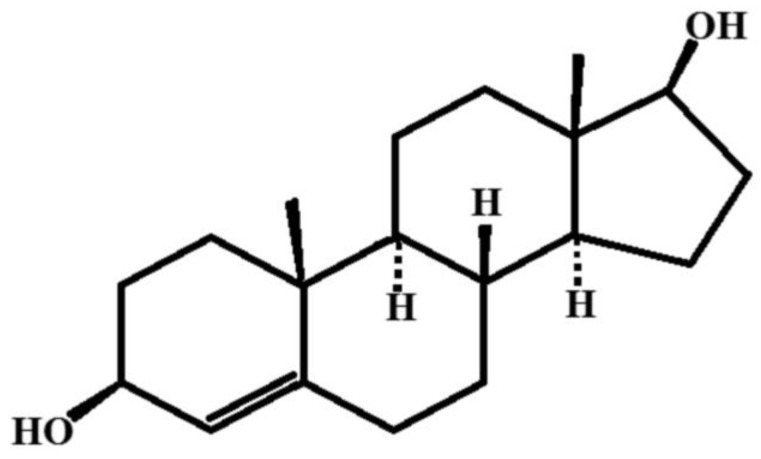
The structural formula is 5-Androstenediol-3β,17β.

5-Androstenediol-3β,17β is a natural hormone with pronounced immune stimulatory and radio protective properties. The chemical formula of the compound is presented in Figure 9.

To produce nanosized particles of 5-Androstenediol-3β,17β using cryochemical methods, a technique involving sublimation in a carrier gas flow combined with low-temperature condensation on the cooled support surfaces was used [68]. This process resulted in obtaining a nanosized monohydrate of 5-Androstenediol-3β,17β. Thin-layer chromatography data indicated that the impurity content in both the original and cryochemically modified samples was less than 0.5%. X-ray phase analysis (XRF) (Rigaku D/MAX-2500, Rigaku, Tokyo, Japan) conducted on the cryochemically modified 5-Androstenediol-3β,17β revealed that its crystallographic structure matched the known structure listed in the Cambridge Crystallographic Database, with the following parameters: a = 6.250 Å, b = 12.143 Å, c = 23.440 Å, α = β = γ = 90°, V = 1779.0 Å^3^, Z = 4, ρvych = 1.152 mg/m^3^, spatial symmetry group P212121. In terms of particle shape and size, the cryochemically modified 5-Androstenediol-3β,17β exhibited a high degree of monodispersity, with drug particles appearing as elongated sticks with rounded ends and an average longitudinal size of 219 ± 9 nm [68].

Androstenediol has the potential to convert into estrogens, primarily in ovaries, but also in adipose tissue. It can also be converted to testosterone. Increased testosterone production from Androstenedione, particularly in the presence of an androgen-producing tumor, often leads to hirsutism and virilization. In the USA, preparations based on this hormone are commonly used by men to increase testosterone levels.

### 6.2. Piroxicam

**Figure 10:**
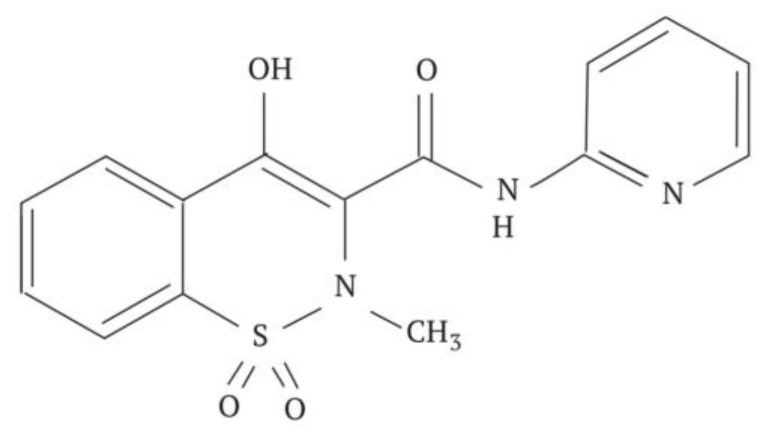
Structural formula of Piroxicam.

Piroxicam is a potent non-steroidal anti-inflammatory drug belonging to the oxime class. It is commonly used to treat conditions such as rheumatoid osteoarthritis, primary dysmenorrhea, postoperative pain, and as an analgesic, especially in cases of complex inflammatory processes. The structural formula of Piroxicam is depicted in Figure 10.

The production of nanosized powders of Piroxicam was achieved using a method involving dynamic sublimation in a heated carrier gas flow followed by low-temperature condensation of the drug substance’s molecular vapor from the gas phase. Analysis of the obtained data confirms that cryochemically modified Piroxicam exists in the pure crystal form III, which is thermodynamically metastable. This form has been recognized for over thirty years, but its crystallographic structure was recently decoded [58]. The crystallographic parameters include a space group of P-1, with a = 8.0106(17) Å, b = 10.080(2) Å, c = 10.519(3) Å; α = 81.215(9)°, β = 69.330(5)°, and γ = 69.827(6)°. The average particle size of cryochemically modified Piroxicam is approximately 300 ± 30 nm [69].

Upon heating, the nanoform of the polymorphic modification Form III of Piroxicam undergoes successive transformations: At a temperature of 125 °C, there is particle enlargement leading to the formation of needle-like crystals characteristic of the polymorphic modification Form II, visible under an optical microscope. At 160 °C, the needle crystals disappear, giving rise to cubic crystals characteristic of the thermodynamically stable Form I.

### 6.3. Phenazepam

**Figure 11:**
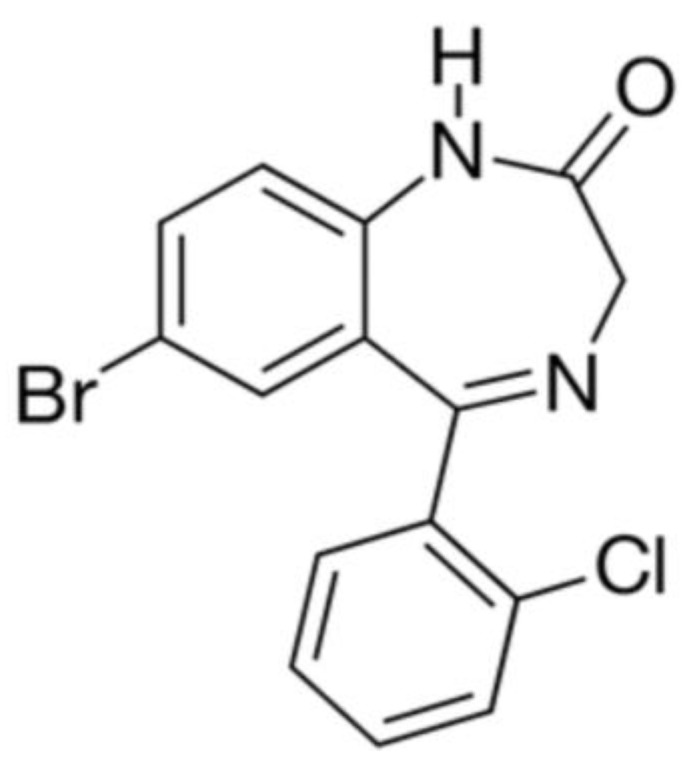
The structural formula of phenazepam.

Phenazepam is a highly effective anxiolytic drug. The chemical structure of the compound is shown in Figure 11.

**Figure 12:**
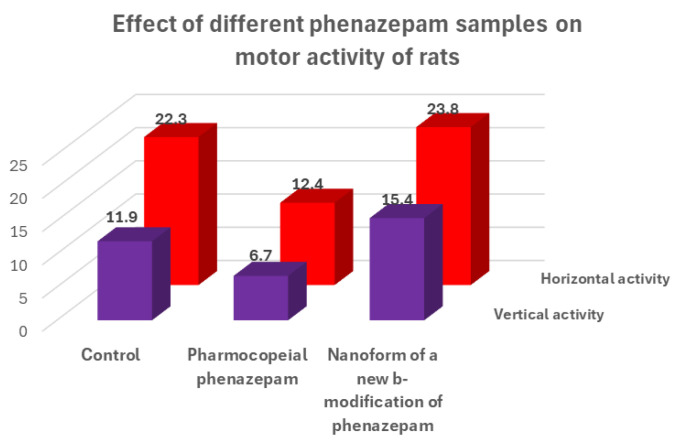
Results of biological tests of different forms of phenazepam on the sedative effect of test animals.

For the cryochemical modification of Phenazepam, a method involving sublimation in a carrier gas flow combined with low-temperature condensation was used [70]. The identity of the composition of the original and cryochemically modified Phenazepam was confirmed by nuclear magnetic resonance (1H, 13C NMR), thin-layer chromatography, and UV spectroscopy. X-ray phase analysis performed on a Rigaku D/MAX-2500 diffractometer (Rigaku, Tokyo, Japan) under CuKα radiation of the initial and cryomodified Phenazepam revealed the formation of a new crystalline β-modification of 7-bromo-1,3-dihydro-5-(2-chlorophenyl)-2H-1,4-benzodiazepin-2-one. The crystal lattice parameters obtained for the new structure are the following: type—monoclinic, with a = 14.792(5) Å, b = 11.678(3) Å, c = 8.472(2) Å, β = 93.677(19)°, V 3 = 1460.4(7) Å^3^ *ρ*_calc_ = 1.59 g/cm^3^, Z = 4 C_15_H_10_N_2_OClBr, space group P21/s. Comparison of the obtained results with those known from the literature indicates that the substance obtained from pharmacopeial Phenazepam is a new crystalline modification of 7-bromo-1,3-dihydro-5-(2-chlorophenyl)-2H-1,4-benzodiazepin-2-one, named by us as the β-modification, with its individual crystal lattice parameters. Detailed data on the new β-modification of Phenazepam have been submitted into the Cambridge Crystallographic Database. The particle diameter was determined by microphotographs obtained on a scanning electron microscope JSM 6380 LA at magnifications of 1000–20,000. The particle diameter of initial Phenazepam ranges from 10 to 120 μm, while that of cryomodified Phenazepam is between 50 and 300 nm depending on the production conditions. The particle sizes of cryomodified Phenazepam estimated from X-ray spectra and surface area data are also in the above range. Tests conducted on glial cells of rat C6 showed that the nanoform of Phenazepam exhibits lower toxicity (Figure 12). 

The tests also revealed that cryochemically modified Phenazepam exhibits an increased anxiolytic (anti-anxiety) activity and reduced sedative effect. A multiple reduction in adverse myorelaxant action was observed, with the ED_50_ dose for the Phenazepam nanoform being approximately five times higher than the conventional form. Biological tests on rats indicated that the composition based on the new crystalline β-modification of Phenazepam displayed enhanced anxiolytic activity and significantly reduced sedative effects.

### 6.4. Carvedilol

**Figure 13:**
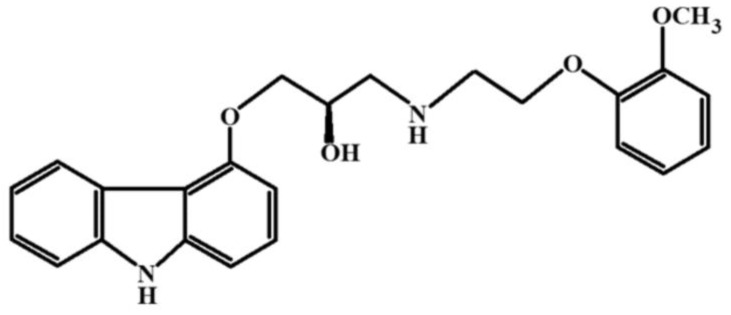
Structural formula of Carvedilol.

Carvedilol, a cardiovascular drug classified as an α- and β-adrenoreceptor blocker, is represented by the chemical formula in Figure 13.

The cryochemical modification of Carvedilol involved sublimation in a carrier gas flow followed by low-temperature condensation of the drug molecular vapor, resulting in an amorphous powder. Analysis using nuclear magnetic resonance methods (13C NMR, 1H NMR) confirmed the identity of the initial Carvedilol, and the obtained X-ray amorphous powder was established. X-ray phase analysis, IR spectroscopy studies, and differential scanning calorimetry were conducted to determine the structural and thermal properties of cryochemically modified Carvedilol. According to the data of X-ray phase analysis, the cryomodified substance is X-ray amorphous. The biological activity of amorphous Carvedilol was tested on two groups of rats, which were administered the original crystalline and amorphous cryomodified Carvedilol. Systemic arterial pressure was recorded through implanted catheters. The amorphous form of Carvedilol was found to increase the inter-pulse interval in animals, possibly through the blockade of β_1_-adrenoreceptors [71].

### 6.5. Gentamicin Sulfate

**Figure 14:**
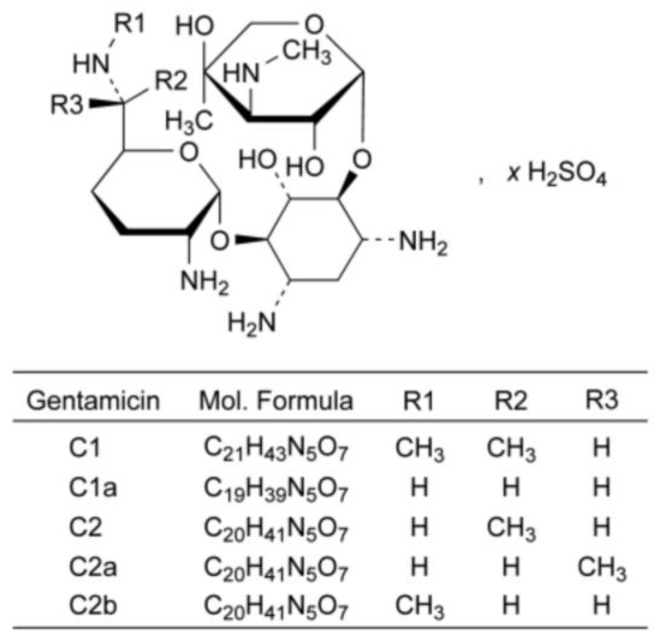
Structural formula of gentamicin sulfate.

Gentamicin sulfate is a natural antibiotic derived from *Micromonospora purpurea* (Figure 14). In contrast to the previously discussed examples, cryochemical modification of the natural antibiotic Gentamicin sulfate allows changing its particle size, but not its crystal structure.

The initial substance of gentamicin sulfate was subjected to cryochemical modification using freeze-drying of the frozen solution. The ^1^H NMR spectra of cryomodified Gentamicin sulfate obtained by the freeze-drying method were in accordance aligned with the literature data, and IR spectra of the original and cryomodified forms were identical. Both the original pharmacopeial Gentamicin sulfate and the cryomodified preparation were X-ray amorphous. However, the specific surface area and the average particle size calculated from the obtained data were different for the original pharmacopeial (1.6 m^2^/g, 2884 nm) and cryomodified (42 m^2^/g, 110 nm) Gentamicin sulfate. The high dispersity of the obtained Gentamicin sulfate powder was also confirmed by TEM microphotographs, from which it can be concluded that cryochemical modification by the method of cryogenic freeze drying leads to the formation of nanoparticles of Gentamicin sulfate with the size of 50–350 nm. Biological testing of the obtained samples of Gentamicin sulfate regarding the growth suppression zone of bacterial cells *S. aureous 244* after 24 h of incubation showed a slight increase in the antibacterial activity of the cryomodified sample compared to the original pharmaceutical substance [72,73].

Based on the presented literature data, it can be concluded that cryochemical modification method enables the reduction in particle size of various drug substances down to nanosizes and the obtainment of new polymorphic crystalline structures. Decreasing the particle size of drug substances to the nanoscale results in an increase in the total specific surface area of the modified samples. At the same time, their physicochemical properties may undergo changes, potentially leading to alterations in the therapeutic properties of cryochemically modified drugs and therapeutic compositions based on them. In some cases, a reduction in undesirable side effects and drug toxicity can be anticipated.

### 6.6. Dioxidine

**Figure 15:**
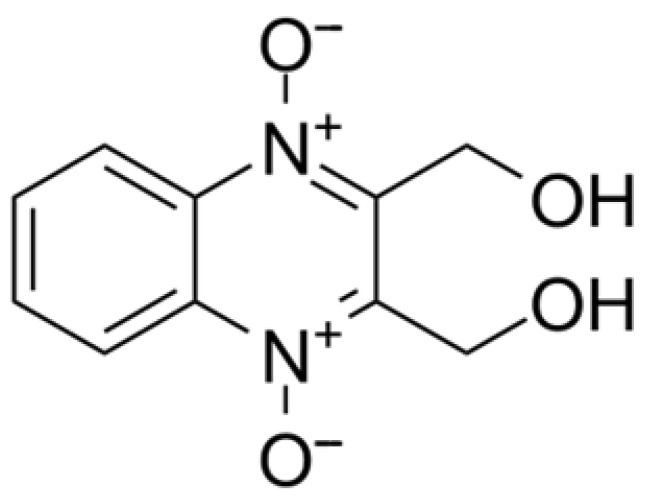
Structural formula of dioxidine.

Dioxidine (2,3-bis-(hydroxymethyl)quinoxaline-N,N,N′-dioxide) is a widely used antimicrobial agent known for effectively inhibiting the growth of numerous Gram-positive and Gram-negative bacteria (Figure 15) [72,73,74,75,76,77,78].

The initial Dioxidine powder underwent cryochemical modification through two methods: freeze-drying of sprayed frozen aqueous solution and low-temperature condensation of molecular vapor from the gas state. The Dioxidine substance corresponding to pharmacopeial standard (FS) 42-2308-97 was used without additional purification. Determination of antibacterial activity of different modifications of Dioxidine was carried out by the disk-diffusion method using pressed tablets and filter paper disks impregnated with solutions of the original and cryomodified drugs. The bacteria *E.coli 52*, *S.aureus 144, M.cyaneum 98*, and *B.cereus 9* were used as test cultures [74,75].

UV spectra analysis revealed that aqueous solutions of pharmacopeial Dioxidine and those modified by freeze-drying and freeze–low-temperature condensation methods exhibited identical characteristics, namely the peaks near 250 nm (a doublet at 241 and 259 nm). However, X-ray diffraction patterns and interplanar crystal lattice distances calculated for the original pharmacopeial Dioxidine and cryomodified samples obtained by freeze-drying and freeze–low-temperature condensation indicated differences, suggesting a change in the crystalline form of Dioxidine during cryochemical modification [75,76]. Powder X-ray diffraction analysis demonstrated the formation of three polymorphic crystalline phases for the cryomodified form of Dioxidine—two anhydrous phases (triclinic and monoclinic) and one hydrated form [75].

**Figure 16:**
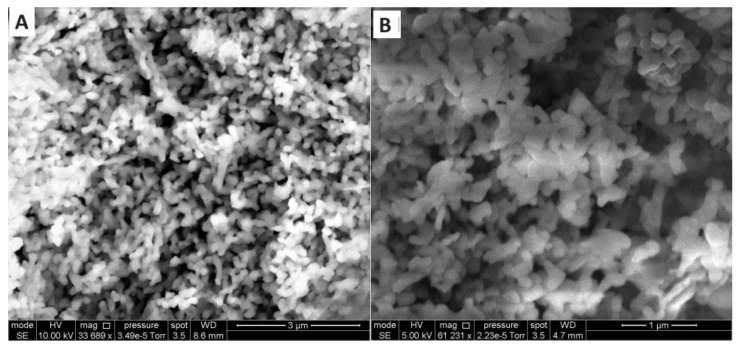
SEM images of cryomodified Dioxidine samples obtained with a gas carrier (CO_2_) flow of 7.0 × 10^16^ molecules s^−1^ cm^−2^ (**A**), with a carrier gas (CO_2_) flow of 7.0 × 10^17^ molecules s^−1^ cm^−2^ (**B**).

IR spectroscopy data indicated that cryomodification led to the creation of new Dioxidine forms characterized by variations in the conformation of Dioxidine molecules and the system of hydrogen bonds that stabilize metastable and stable crystal structures. In the case of pharmacopeial Dioxidine, the vibrational band of the quinoxaline ring is at 1506 cm^−1^, and the C-H vibrational band of the aromatic ring exhibits a peak at 971 cm^−1^ and two doublets at 1117 and 1113, 1152, and 1160 cm^−1^, the C-O-H vibrational band is also doublet at 1280 and 1288 cm^−1^. In case of Dioxidine cryomodified by the freeze-drying method, the vibrational band of quinoxaline ring is at 1510 cm^−1^, the C-H vibrational bands of benzene ring are observed at 975, 113, and 1160 cm^−1^ and the C-O-H vibrational band is found at 1288 cm^−1^. The IR spectra of Dioxidine cryomodified by freeze-drying–low-temperature condensation method differs from Dioxidine modified by the freeze-drying method by the C-O bond vibration band (2236 cm^−1^) of the CO_2_ molecule [76]. The specific surface area was determined by low-temperature argon adsorption and the average particle size of the original (0.7 m^2^/g, 5700 nm) and cryomodified by freeze-drying (21.3 m^2^/g 190 nm) and low-temperature condensation (47 m^2^/g, 85 nm) dioxidine was obtained. The decrease in the size of drug particles during cryomodification is confirmed by microphotographs obtained by SEM [76] (Figure 16).

The freeze-dried Dioxidine was active against *E.coli 52*, *S.aureus 144*, *M.cyaneum 98*, and *B.cereus 9.* After 48 h of incubation, the antibacterial activity of the cryomodified freeze-dried preparation was higher for all the bacterial strains studied (Table 1).

The dimensional and crystallographic parameters of some drugs nanoforms obtained by cryochemical modification methods are summarized in Table 2.

## 7. Alternative Methods for Producing Medicinal Nanoparticles 

The hydrophobic drugs silymarin, β-carotene, and butylated hydroxytoluene have been successfully prepared in nanoparticulate form (~100 nm in diameter) using the *nanoporous membrane extrusion method* [79]. This technique involves pumping the drug in a solvent phase through a nanoporous membrane into a receiver solution. The membrane used for drug modification was 60 µm-thick and had 200 nm cylindrical pores at the face of the membrane in contact with the receiver solution, and 20 nm pores in contact with the feed solution. The obtained nanoparticles were amorphous and showed an improved dissolution profile compared with untreated drug powders. For instance, more than 90% of silymarin nanoparticles were dissolved in 8 hours, whereas only about 25% of the bulk silymarin precursor dissolved within the same timeframe. The improved dissolution profile could be attributed to the amorphous nature of the drug nanoparticles and their increased surface area. 

The *co-precipitation and precipitation methods*, along with milling, are currently used to obtain nanocrystals of medicinal substances for the pharmaceutical industry. These methods were used to obtain nanoparticles of Griseofulvin, Nabilone, and Itraconazole [80]. 

*Spray drying* is a continuous, reproducible, and scalable process for producing dry powders from a fluid material by atomization through an atomizer into a hot drying gas medium, typically air. It could be used for both dehydration process and encapsulating hydrophilic and hydrophobic active compounds within different carriers [81]. In the study [80], spray-drying technology was found to be effective in producing steroid drugs fluorometholone and dexamethasone nanoparticles. Depending on the synthesis conditions, the average size of fluorometholone nanocrystals ranged from 620 to 856 nm, while for dexamethasone, it was between 833 and 1118 nm.

*Pulsed laser deposition* is another method to obtain nanopowders of medicinal substances. Phenytoin and indomethacin were irradiated [82] with a laser at a wavelength of 266 or 1064 nm by hot pressing to obtain nanoparticles of drugs less than 20 nm in size. An essential requirement is that medicinal substances should not decompose during the process.

## 8. Micronization and Properties of Medicinal Substances

Transforming medicinal substances into nanoform allows for an increased dissolution rate, saturation solubility, absorption efficiency by the body, optimal administration methods, and, ultimately enhances treatment effectiveness. For instance, Tadalafil, a phosphodiesterase-5 inhibitor used to treat pulmonary arterial hypertension, exhibited improved dissolution behavior when formulated as a nanocomposition through cryogenic spray drying compared to pure Tadalafil. This formulation enabled a switch in the drug administration route from oral to inhalation, leading to increased drug concentration in the target organ. Micronization facilitated more efficient administration methods and enhanced Tadalafil absorption by the body, thereby boosting treatment efficacy [83].

In targeted drug delivery to the bronchi and lungs, therapeutic drug aerosols are highly effective. The lungs’ thin air–blood barrier, large contact surface area, and numerous capillaries make them an ideal entry point for targeted drug delivery systems. The lung’s lower metabolic activity relative to the liver and gastrointestinal tract along with the ease of administering drug systems are key advantages of this site of drug entry into the body. Particle size plays a critical role in determining the deposition of inhaled aerosols in the lungs, as well as their adsorption and penetration rate into the circulatory system [13,14].

Colloidal nanotechnologies play a vital role in the formation and growth of nanoparticles in emulsions, particularly in the nanoengineering of various biomedical materials [15,16,17]. Nano- and microemulsions are colloidal dispersions of oil in water (or water in oil), stabilized by an interfacial film of surfactants. Nanoemulsions, as dosage forms, offer enormous advantages for drugs with solubility, lipophilicity, and bioavailability challenges [18]. However, the use of this method is limited by the necessity of surfactants and solvents used in targeted drug delivery systems administered intravenously. Efforts are ongoing to explore the use of microemulsions as templates for nanoengineering hydrophobic drugs [6].

The effect of particle size on the amount of drug absorbed during colonic delivery was computationally modeled in [19]. The research highlighted that, besides particle size, the effectiveness of colonic drug administration is influenced by the solubility of the medicinal substance and the digestive cycle.

Naringenin, a citrus flavonoid with significant biological actions, underwent particle size reduction using supercritical technologies [84]. In vitro dissolution rate assays of naringenin microparticles confirmed that the micronized preparation exhibited a significantly higher dissolution rate than the original form. Its application in an in vitro mouse model has shown positive symptoms in schizophrenia. 

Poor water solubility and low oral bioavailability of mangiferin was improved by means supercritical antisolvent nanonization technology. Submicron particles of the drug exhibited a higher solubility approximately 4.26, 2.1, and 2.5 times higher than the solubility of the bulk substance in water, artificial gastric juice, and artificial intestinal juice, respectively. The dissolution rate of the nanosized drug was also higher than the dissolution rate of the unmodified drug. In addition, the in vivo bioavailability of the resulting particles and their antioxidant capacity were significantly higher than those of the original bulk preparation [85]. Micronization carried out using supercritical technologies increases the bioavailability of curcumin [86].

## 9. Conclusions

In recent decades, different scientific groups have proposed intriguing examples of nanosized drug particle formation. This paper offers an overview of processes for producing pharmaceutical nanoparticles through both “top-down” and “bottom-up” approaches. Due to their high specific surface area, drug nanoparticles strongly differ from their bulk precursors in terms of dissolution rate, saturation solubility, ability to overcome biological barriers, and interactions with biological molecules. Changes in particle size often coincide with alterations in the crystal structure of drug nanoforms or the formation of amorphous phases. In many cases, this leads to enhanced effectiveness in biomedical applications and therapeutic effect of nanosized drugs particles compared to their bulk precursors. Numerous studies of drug micronization have shown that it contributes to increased solubility, dissolution rate, bioavailability, and therapeutic efficiency. While traditional methods like grinding in mills and electro-spraying were initially used for micronizing medicinal substances, recent years have seen a preference for supercritical and low-temperature technologies. 

One promising and rapidly advancing method for nanonization and micronization of medicinal substances involves supercritical fluidic technologies. These technologies leverage the extreme increase in solubility in supercritical solvents, rapid precipitation, and crystallization using anti-solvents. By adjusting experimental conditions, this micronization technique enables the production of medicinal nanoparticles devoid of toxic solvents by varying the experimental conditions, this technique allows to obtain medicinal particles in a wide range of shapes and sizes controlled by experimental parameters—temperature and pressure in the reactor, and varying the chemical nature of supercritical fluids.

Another powerful method for obtaining nanoforms of drug substances is cryochemical modification and the production of nanosized particles of various drugs. One of the variants of this method is based on the sublimation or evaporation of a drug substance under high-vacuum conditions and the inclusion of the resulting vapors in a flow of inert gas, followed by low-temperature condensation of a molecular beam of a drug substance from the gas phase on a cooled surface of the cryostat. Another method of cryogenic drug substances’ micronization is spray freeze drying. These technologies make it possible to obtain nanoparticles of medicinal compounds without the use of toxic organic solvents, with controlled particle size, shape, and crystal structure varying experimental parameters—temperatures of drug substance evaporation (sublimation) and vapor condensation, residual pressure and geometry of the cryostat, inert carrier gas nature, and flow rate. Cryochemical methods for obtaining nanoforms of drug substances make it possible to control the size of the resulting drug nanoparticles with a narrow size distribution; their crystal structure and morphology can be changed, including the formation of new polymorphic crystalline modifications, which leads to the production of drugs with the desired and improved biomedical properties. 

The development of mathematical models to describe unique physicochemical processes during medicinal substance particle formation and the optimization of the experimental parameters will aid in obtaining medical nanoparticles with desired dimensional and structural properties, high stability, and improved therapeutic efficiency.

## Figures and Tables

**Table 1:** Diameters of zones of inhibition (ZOI) of growth of *E.coli 52*, *S.aureus 144*, *M.cyaneum 98*, and *B.cereus 9* around tablets of original pharmacopeial and cryomodified dioxidine after 48 h of incubation [77].

Bacterial Strains	Diameter of Zones of Bacterial Growth Inhibition (ZOI) around Initial Dioxidine Tablets, mm	Diameter of Zones of Bacterial Growth Inhibition (ZOI) around Cryodioxidine Tablets, mm
*E.coli 52*	33.3 ± 0.6	37.7 ± 1.0
*S.aureus 144*	19.7 ± 1.2	29.7 ± 1.2
*M.cyaneum 98*	25.3 ± 1.2	36.0 ± 0.6
*B.cereus 9.*	29.3 ± 1.0	31.0 ± 1.2

**Table 2:** Nanoforms of some medicinal substances, obtained by cryochemical methods and their properties.

Pharmaceutical Substance	Method	Crystallographic Parameters	Size, nm	Reference
5-Androstenediol- 3β,17β	Sublimation in a carrier gas flow—low-temperature condensation	a = 6.250 Å, b = 12.143 Å, c = 23.440 Å, α = β = γ = 90°, V = 1779.0 Å^3^, Z = 4, P212121	219 ± 9	[68]
Piroxicam	Sublimation in a carrier gas flow—low-temperature condensation	P-1; a = 8.0106(17) Å, b = 10.080(2) Å, c = 10.519(3) Å; α = 81.215(9)°, β = 69.330(5)°, γ = 69.827(6)°	300 ± 30	[69]
Phenazepam	Sublimation in a carrier gas flow—low-temperature condensation	a = 14.792(5) Å, b = 11.678(3) Å, c = 8.472(2) Å, β = 93.677(19)°, V 3 = 1460.4(7) Å^3^, Z = 4, P21/s	50–300	[70]
Carvedilol	Sublimation in a carrier gas flow—low-temperature condensation	Amorphous	50–300	[71]
Gentamicin sulfate	Spray cryogenic freeze drying	Amorphous	110 ± 11	[72,73]
Dioxidine	Sublimation in a carrier gas flow—low-temperature condensation/ Spray cryogenic freeze drying	Triclinic/monoclinic/orthorhombic phase depends on the conditions of obtaining	85/190	[74,75,76,77,78]

## Data Availability

Data sharing is not applicable.
